# Genome-Wide Identification and Expression Profiling of 2OGD Superfamily Genes from Three *Brassica* Plants

**DOI:** 10.3390/genes12091399

**Published:** 2021-09-10

**Authors:** Ding Jiang, Guangguang Li, Guoju Chen, Jianjun Lei, Bihao Cao, Changming Chen

**Affiliations:** 1 Key Laboratory of Biology and Germplasm Enhancement of Horticultural Crops in South China, Ministry of Agriculture, College of Horticulture, South China Agricultural University, Guangzhou 510642, China; jiangding92@gmail.com (D.J.); gjchen@scau.edu.cn (G.C.); jjlei@scau.edu.cn (J.L.); caobh01@163.com (B.C.); 2 Guangzhou Institute of Agriculture Science, Guangzhou 510335, China; liguangguang2019@163.com

**Keywords:** *Brassica* plants, 2-oxoglutarate and Fe(II)-dependent dioxygenase, genome-wide, phylogenetic analysis, gene expression analysis

## Abstract

The 2-oxoglutarate and Fe(II)-dependent dioxygenase (2OGD) superfamily is the second largest enzyme family in the plant genome, and its members are involved in various oxygenation and hydroxylation reactions. Due to their important biochemical significance in metabolism, a systematic analysis of the plant 2OGD genes family is necessary. Here, we identified 160, 179, and 337 putative 2OGDs from *Brassica rapa*, *Brassica oleracea*, and *Brassica napus*. According to their gene structure, domain, phylogenetic features, function, and previous studies, we also divided 676 2OGDs into three subfamilies: DOXA, DOXB, and DOXC. Additionally, homologous and phylogenetic comparisons of three subfamily genes provided valuable insight into the evolutionary characteristics of the 2OGD genes from *Brassica* plants. Expression profiles derived from the transcriptome and Genevestigator database exhibited distinct expression patterns of the At2OGD, Br2OGD, and Bo2OGD genes in different developmental stages, tissues, or anatomical parts. Some 2OGD genes showed high expression levels in various tissues, such as callus, seed, silique, and root tissues, while other 2OGD genes were expressed at very low levels in other tissues. Analysis of six Bo2OGD genes in different tissues by qRT-PCR indicated that these genes are involved in the metabolism of gibberellin, which in turn regulates plant growth and development. Our working system analysed 2OGD gene families of three *Brassica* plants and laid the foundation for further study of their functional characterization.

## 1. Introduction

In the plant kingdom, oxidative enzymes participate in catalysing many different reactions of metabolism and this suite of enzymes includes 2-oxoglutarate and Fe(II)-dependent dioxygenases (2OGDs) [1]. 2OGDs are non-haem iron-containing proteins, and as soluble proteins, they are localized in the cytosol [1]. 2OGDs require 2-oxoglutarate (2OG) and molecular oxygen as co-substrates and ferrous iron Fe(II) as a cofactor to catalyse the oxidation of a substrate with concomitant decarboxylation of 2OG to form succinate and carbon dioxide (R + 2OG + O_2_ → R–OH + succinate + CO_2_) [1,2,3,4,5,6]. 2OGDs consist of a non-haem dioxygenase in morphine synthesis N-terminal (DIOX_N) [7] in the N-terminal region and a 2OG-Fe(II) oxygenase superfamily (2OG-FeII_Oxy) domain in the C-terminal region [8,9].

In *Arabidopsis thaliana* L. Heynh., according to phylogenetic function, the 2OGD superfamily is classified into three subfamilies: DOXA, DOXB, and DOXC [1]. The DOXA class contains 14 2OGD members [1], and they function in N-methyl hydroxylation and as DNA repair proteins [10,11]. The DOXB class contains 14 prolyl 4-hydroxylases (P4Hs), and they participate in the modification of proline hydroxylation of cell wall proteins [12]. The DOXC class is the largest class and is composed of 100 2OGD members involved in the biosynthesis of secondary metabolites, such as 1-aminocyclopropane carboxylic acid oxidases (ACOs) [13], anthocyanidin synthases (ANSs) [14,15,16], GA 2-oxidases (GA2oxs), GA 20-oxidases (GA20oxs) [17], flavanone 3-hydroxylases (F3Hs) [18], dioxygenases for auxin oxidation (DAOs) [19,20,21], and glucosinolate biosynthesis (AOPs) [22,23,24,25]. The catalytic conversion of 2OGDs in plant metabolism is diverse [1]. In primary metabolism, 2OGDs have established roles in DNA repair, histone demethylation, post-translational modification, epigenetics, and plant growth regulator activation and catabolism. In secondary metabolism, 2OGDs take part in multiple pathways and show as much functional diversity as cytochrome P450 monooxygenases (CYPs) [8,26]. Therefore, the 2OGD superfamily is very important, and it is necessary to study this 2OGD superfamily.

With the advancement of nucleotide sequencing, nucleotide sequence data have enabled genome-wide identification, mapping, and characterization of candidate gene families in many important plants. For example, the approximate arrays of 130 2OGD genes in *A. thaliana* [27], 114 in *Oryza sativa* L. [28], 142 in *Picea abies* L. H. Karst. [29], and 132 in *Salvia miltiorrhiza* Bunge [30] have been identified. According to the number of 2OGD genes in *A. thaliana*, we can predict a plethora of reactions and roles for 2OGDs in other plant species. Given the known importance of 2OGDs in plant metabolism, the continued functional characterization of 2OGDs in economically important plants is essential. With the recent development of the *Brassica* Plant Genome Project and the number of genes identified and classified into each of the gene superfamilies, the prediction of the functionality of multiple genes has become a concern. The present study aimed to identify 2OGD genes on a genome-wide scale in *Brassica napus* L., *Brassica oleracea* L. and *Brassica rapa* L. and provide insights into their roles in evolutionary history, DNA repair, antibiotic biosynthesis, collagen biosynthesis, endogenous hormone synthesis/metabolism, and stress responses.

## 2. Materials and Methods

### 2.1. Genome-Wide Investigation of 2OGD Genes in Three Brassica Plants

The *A. thaliana* 2OGD sequences were from a known reference [1]. The *A. thaliana* 2OGD protein sequences were downloaded from the NCBI database (http://www.ncbi.nlm.nih.gov/protein/, accessed on 1 January 2018). A total of 130 *A. thaliana* 2OGD protein sequences were used as queries in BLASTp searches with the Genome database, including *B. napus*, *B. rapa*, and *B. oleracea*, using a cut-off e-value of 1.0 × 10^−10^. The same gene IDs were removed, and the sequences with lengths less than 400 bp were discarded. The 130 available At2OGD sequence data are listed in Supplementary Table S1a. The retrieved sequences were then automatically checked for the DIOX_N and 2OG-FeII_Oxy superfamily domain in the Pfam server (http://pfam.xfam.org/search#tabview=tab1, accessed on 1 January 2018) or SMART server (http://smart.embl-heidelberg.de/, accessed on 1 January 2018). The proteins lacking complete DIOX_N or 2OG-FeII_Oxy domains were then discarded.

### 2.2. Sequences and Chromosomal Location Analyses

The Compute pI/Mw tool on the ExPASy server (http://web.expasy.org/compute_pi/, accessed on 1 January 2018) was employed to predict the theoretical isoelectric point (pI) and molecular weight (Mw) for each 2OGD protein. The TBtools tool [31] was used to display precise gene locations on each chromosome and using the input coding sequences and corresponding genomic sequences, analyse the gene chromosomal location of 2OGDs.

### 2.3. Gene Structural Determination and Subcellular Location

Conserved domains in 2OGD proteins were identified using an online search in Pfam [32] (Suite version, http://pfam.xfam.org/search#tabview=tab1, accessed on 1 January 2018). We used HMMER to perform a hidden Markov Model-based domain search against all amino acid sequences of *Brassica* plants [33] with a cut-off gathering threshold. The TBtools tool [31] was used to generate a domain map of the 2OGD protein sequence with the Pfam search results. The CELLO v.2.5 server (http://cello.life.nctu.edu.tw/, accessed on 1 January 2018) was used to predict the subcellular localization of the 2OGD proteins [34].

### 2.4. Sequence Homologous and Phylogenetic Analyses of 2OGD

All identified 2OGD protein sequences from *A. thaliana*, *B. napus*, *B. rapa*, and *B. oleracea* were pooled into MEGA-X [35], and multiple sequence alignments were performed. The bootstrap method was used to build maximum parsimony trees and the pairwise deletion of gaps/missing data with 1000 replications. The phylogenetic trees were built using the maximum parsimony method with Subtree-Pruning-Regrafting (SPR). Statistical support for the nodes on the maximum parsimony tree was evaluated by bootstrap analysis with 1000 replicates. All reconstructed trees were unrooted.

### 2.5. Gene Expression Analysis in Different Tissues

The expression data of the 2OGD genes at developmental stages were from *A. thaliana* eFP Browser (http://bar.utoronto.ca/efp/cgi-bin/efpWeb.cgi, accessed on 1 April 2018). The RNA-Seq data (GSE30795) from two founder accessions of *A*. *thaliana* were selected to evaluate the expression of the predicted 2OGDs in different tissues, for example: seedling, root, and floral bud tissue. The RNA-Seq data (GSE43245) from *B. rapa* and data (GSE42891) from *B. oleracea* were selected to investigate the expression patterns of the 2OGD genes in different tissues, such as the callus, bud, flower, leaves, roots, silique, and stem. A heatmap of the expression data was obtained using the distance function (Euclidean) and hierarchical clustering (Average) on the TBtools tool [31]. To compare tissue-specific expression data of *B. rapa* and *B. oleracea*, we analysed the RNA-Seq and microarray data of *A. thaliana* for expression of 130 2OGDs across 25 various tissues and 105 anatomical parts using the Genevestigator database (https://genevestigator.com/gv/, accessed on 1 April 2018).

### 2.6. Expression Analysis by qRT-PCR

Our research group mainly focused on the molecular biology study of Chinese kale (*Brassica oleracea* var. *chinensis* Lei). Therefore, we only performed RT-qPCR on six genes related to GA metabolism in different tissues of Chinese kale. The Chinese kale was planted in plastic pots and collected according to different tissues when the plants grew to inflorescences up to the apical leaves [36]. The plants were divided into 11 parts (further called “tissues” in this article) as shown in Supplementary Figure S1 [34]. All samples were cleaned and washed with double distilled water. Pieces of tissues from more than 5 plants were pooled into one sample. Samples of each tissue were ground into fine powders in liquid nitrogen and total RNA was extracted using FastPure^®^ Plant Total RNA Isolation Kit (Vazyme Biotech Co., Ltd., Nanjing, China), according to the manufacturer’s instructions. Quantitative real-time RT-PCR was carried out using a SYBR Green I Mix (TaKaRa, Dalian, China) as previously described [37]. Gene-specific and Actin-specific (reference gene) primers were designed for the six Bo2OGD genes of Gibberellin metabolism-related and Actin (Supplementary Table S2). Triplicate technical replicates in quantitative PCR experiments were performed for each sample. Analysis of the relative gene expression data was con-ducted using the 2^−ΔΔCt^ method [38].

## 3. Results

### 3.1. Genome-Wide Identification of 2OGD Members in Brassica Plants

We identified 676 putative 2OGDs in three *Brassica* species, including 160 2OGDs from *B. rapa*, 179 from *B. oleracea*, and 337 from *B. napus*, with sequence similarities of 78.21~ 94.33% with the *A. thaliana* 2OGDs (Supplementary Table S3). The Pfam online tool was used for the identification of 2OGDs and produced consistent results in terms of the DIOX_N (Pfam Accession: PF14226.5) or 2OG-FeII_Oxy (Pfam Accession: PF03171.19, PF13532.5, and PF13640.5) domains. These predicted genes all contained these domains. The list of all putative 2OGD genes were presented in Supplementary Table S1. The CDS of these genes was 441 to 1806 bp in length, and the longest 2OGD (BnaA06g09520D from *B. napus*) included eight exons. The length of the 2OGD proteins varied from 136 (BnaC04g41480D) to 979 amino acids (BnaA06g09520D), with corresponding Mw ranged from 15.3 kDa to 65.9 kDa, and the theoretical pI ranged from 4.47 (Bo6g030830) to 9.30 (Bo9g104660) (Supplementary Table S1). In *A. thaliana*, the 2OGD proteins were predicted to be located in the cytoplasm (91 2OGDs), nucleus (24 2OGDs), chloroplast (1 2OGDs), extracellular space (6 2OGDs), mitochondria (4 2OGDs), plasma membrane (3 2OGDs), and peroxisomes (1 2OGDs). In *B. napus*, the 2OGD proteins were predicted to be located in the cytoplasm (223 2OGDs), nucleus (57 2OGDs), chloroplast (12 2OGDs), extracellular space (20 2OGDs), mitochondria (13 2OGDs), and plasma membrane (12 2OGDs). In *B. rapa*, the 2OGD proteins were predicted to be located in the cytoplasm (109 2OGDs), nucleus (25 2OGDs), chloroplast (6 2OGDs), extracellular space (9 2OGDs), mitochondria (6 2OGDs), and plasma membrane (5 2OGDs). In *B. oleracea*, the 2OGD proteins were predicted to be located in the cytoplasm (121 2OGDs), nucleus (30 2OGDs), chloroplast (7 2OGDs), extracellular space (10 2OGDs), mitochondria (6 2OGDs), and plasma membrane (5 2OGDs) (see Supplementary Table S4 for more details). Most of the 2OGD proteins were predicted to be located in the cytoplasm or nucleus.

### 3.2. Chromosomal Locations of the 2OGD Gene Family

**Figure 1:**
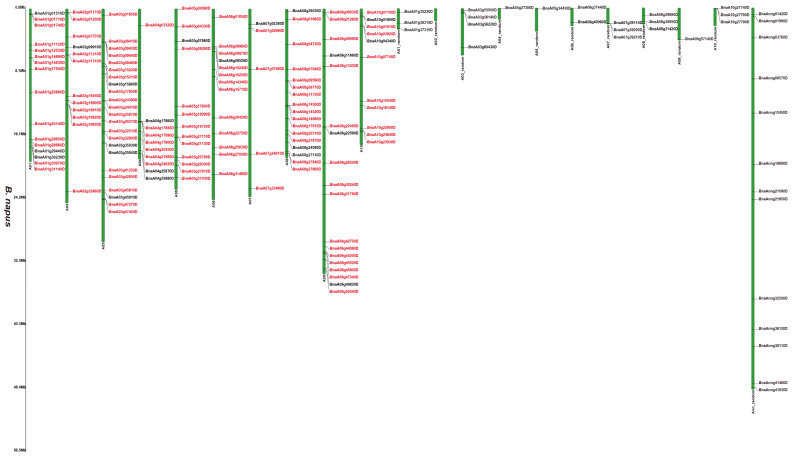

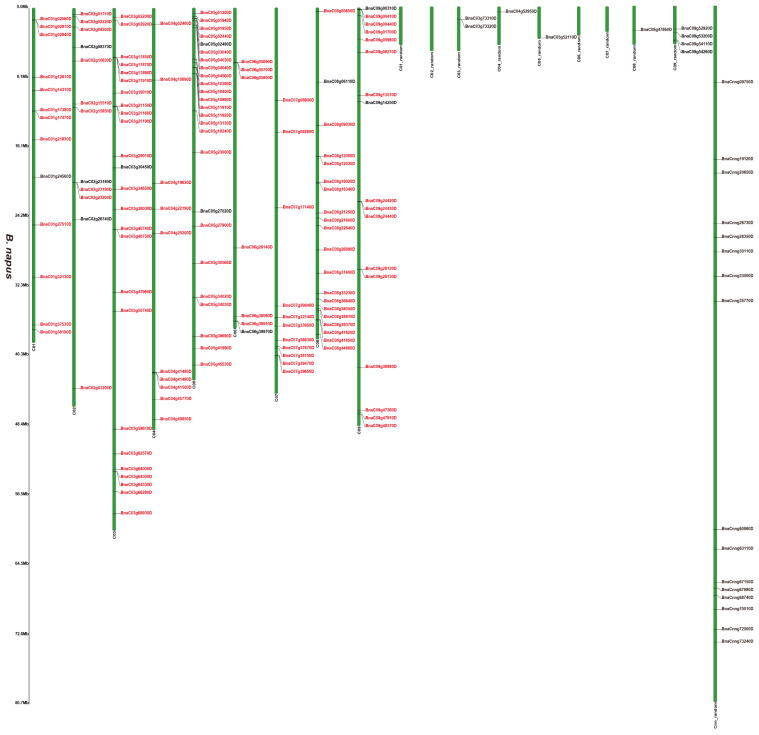
Chromosomal distribution of putative 2OGDs in *B. napus*. Individual 2OGD positions are shown in Mbs along the chromosome scale. The red label represents the homologous gene.

**Figure 2:**
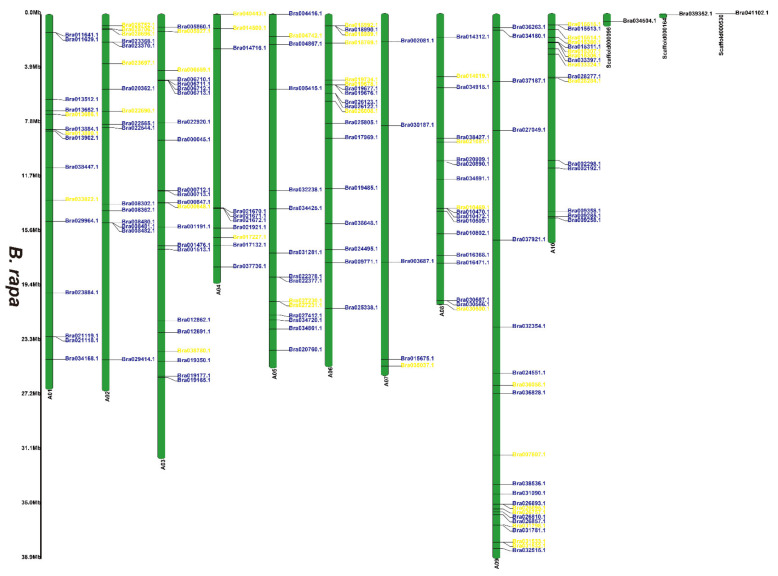
Chromosomal distribution of putative 2OGDs in *B. rapa*. Individual 2OGD positions are shown in Mbs along the chromosome scale. The blue label represents the retained gene, the yellow label represents the lost gene.

**Figure 3:**
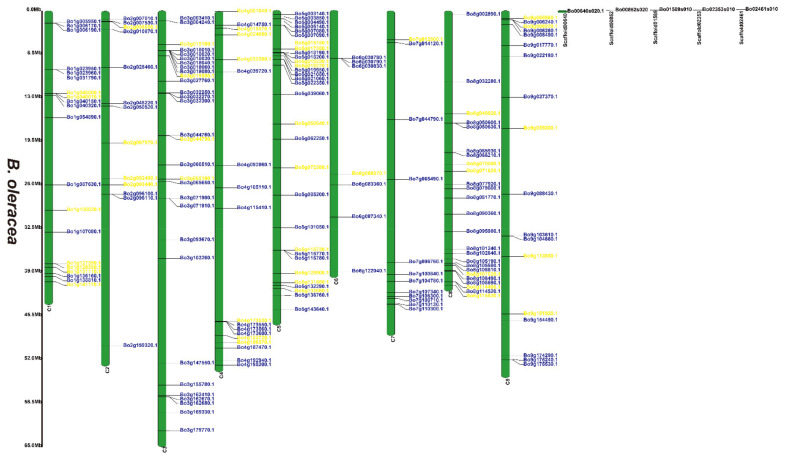
Chromosomal distribution of putative 2OGDs in *B. oleracea*. Individual 2OGD positions are shown in Mbs along the chromosome scale. The blue label represents the retained gene, the yellow label represents the lost gene.

Supplementary Figure S2 and Figure 1, Figure 2 and Figure 3 illustrates 2OGD gene localization on chromosomes of *A. thaliana* and *Brassica* species. The *A. thaliana* chromosomes 1~5 contained 43, 15, 23, 24, and 25 2OGDs, respectively (Supplementary Figure S2). Some 2OGD genes were located very close to a specific location on the chromosomes. The presence of clusters of 2OGD genes from the same clade is strongly suggestive of very recent tandem gene duplication events. The *Brassica* species chromosomes contained at least one 2OGD gene (Figure 1, Figure 2 and Figure 3). The AA chromosomes of 1~10 *B. napus* contain 16, 12, 23, 10, 13, 13, 5, 17, 18, and 11 2OGDs, respectively. The CC chromosomes of 1~9 of *B. napus* contain 13, 12, 25, 10, 25, 7, 11, 20, and 17 2OGDs, respectively. Some 2OGD genes were located at specific positions on the chromosome to form gene clusters. The presence of a 2OGD gene cluster was accompanied by tandem gene duplication events, such as the tandem gene duplication BnaA03g09460D, BnaA03g09440D, BnaA03g09430D, and BnaA03g09410D (Figure 1). The *B. rapa* chromosomes 1~10 contain 15, 16, 22, 10, 15, 18, 5, 18, 22, and 16 2OGDs, respectively (Figure 2). The *B. oleracea* chromosomes 1~9 contain 20, 13, 30, 18, 31, 7, 12, 25, and 18 2OGDs, respectively (Figure 3). A comparison between *B. rapa* and *B. napus* AA chromosomes or between *B. oleracea* and *B. napus* CC chromosomes revealed that the putative 2OGDs of *B. napus* followed the exact pattern of chromosome involvement as its ancestors *B. rapa* and *B. oleracea* (Figure 1, Figure 2 and Figure 3). In the scaffolds of *B. rapa* and *B. oleracea*, some genes (Bo00640s020.1, Bo00852s020, Bo01589s010, Bo02353s010, Bo02461s010, Bra034504.1, Bra039352.1, and Bra041102.1) could not be located in any chromosome (Figure 2 and Figure 3). In *B. napus*, we found that 59 2OGD genes were mapped to random fragments of different chromosomes (Figure 1). Among the three *Brassica* species, the largest clusters of 2OGDs were located on chromosomes A3, A10, C3, C5, and C8 (Figure 1, Figure 2 and Figure 3). There were also tandem gene repeat events in the three *Brassica* species, for example, BnaC04g41480D-BnaC04g41490D-BnaC04g41500D, Bo3g018010.1-Bo3g018020.1-Bo3g018030.1-Bo3g018040.1, and Bra006710.1-Bra006711.1-Bra006712.1-Bra006713.1. Tandem replication mainly occurred in the region of chromosome recombination, and the members of the gene family formed by tandem replication were usually closely arranged on the same chromosome to form a cluster of genes with similar sequences and similar functions. Therefore, these tandem repeat genes may have the same functions.

We have marked the genes in red labels of *B. napus* (Figure 1) that are homologous genes from ancestral *B. rapa* (Figure 2) and *B. oleracea* (Figure 3). In the process of hybridization and gene replication, the blue labels in *B. rapa* and *B. oleracea* represented these genes were retained in the A or C genomes of *B. napus* during the evolution, and the yellow labels represented these genes were lost in the A or C genome of *B. napus* during the evolution. This allows us to understand more clearly about the relationship between gene copies and species evolution among the three *Brassica* plants. For example, the tandem genes BnaA01g01710D and BnaA01g01740D on chromosome A01 of the *B. napus* A genome were corresponding homologous genes to the tandem genes Bra011641.1 and Bra011639.1 on chromosome A01 of *B. rapa*. The tandem genes BnaC01g02810D and BnaC01g02840D on chromosome C01 of the *B. napus* C genome were the corresponding homologous genes to the tandem genes Bo1g006170.1 and Bo1g006190.1 on chromosome C1 in *B. oleracea*. More than two thirds of genes have very similar chromosomal location distribution in each species. This suggested that two thirds of genes of the ancestral *B. rapa* and *B. oleracea* were retained in the *B. napus*, and one third of genes were lost or differentiated into other genes.

### 3.3. Phylogenetic Analysis and Diversity of 2OGDs

**Figure 4:**
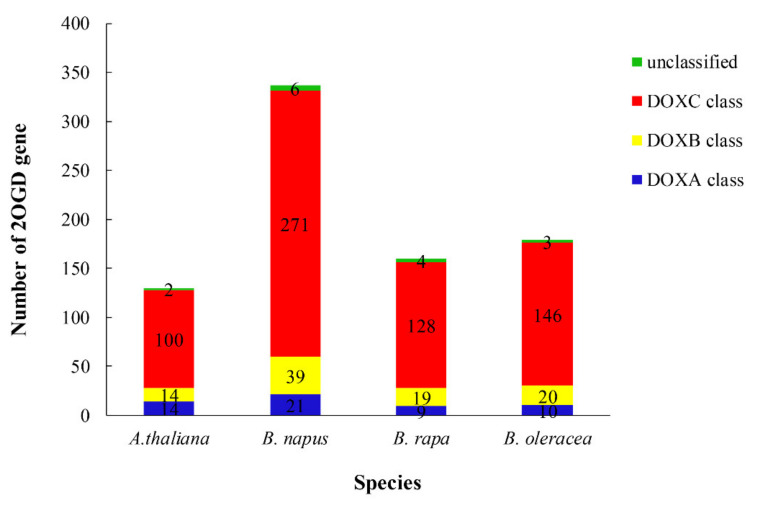
Compositional differences in the 2OGD classes in *Brassica* species and *A. thaliana*. The number of 2OGD genes in the DOXA, DOXB, and DOXC classes is represented by different colours; ‘unclassified’ represents 2OGD genes that belong to none of the classes.

Sequences very similar to the *A. thaliana* 2OGD sequence were placed in the same group. The DOXA, DOXB and DOXC groups were classified, and they have different functions. Figure 4 shows that the DOXC class accounts for the majority of 2OGDsin the three *Brassica* plants. There were six genes of *B. napus*, four genes of *B. rapa,* and three genes of *B. oleracea* that were not yet classified. Twenty-one genes of *B. napus*, nine genes of *B. rapa,* and ten genes of *B. oleracea* belonged to the DOXA class of 2OGDs (Figure 4). Thirty-nine genes of *B. napus*, nineteen genes of *B. rapa,* and twenty genes of *B. oleracea* were classified in the DOXB class (Figure 4). A total of 271 genes of *B. napus*, 128 genes of *B. rapa*, and 146 genes of *B. oleracea* were classified in the DOXC class (Figure 4). The details of each class are shown in Supplementary Table S3. The detailed phylogeny analysis of 2OGD genes between *A. thaliana*, *B. oleracea*, *B. rapa*, and *B. napus* suggests a high correlation between the evolution and a structural organisation of the 2OGD gene family in these species.

**Figure 5:**
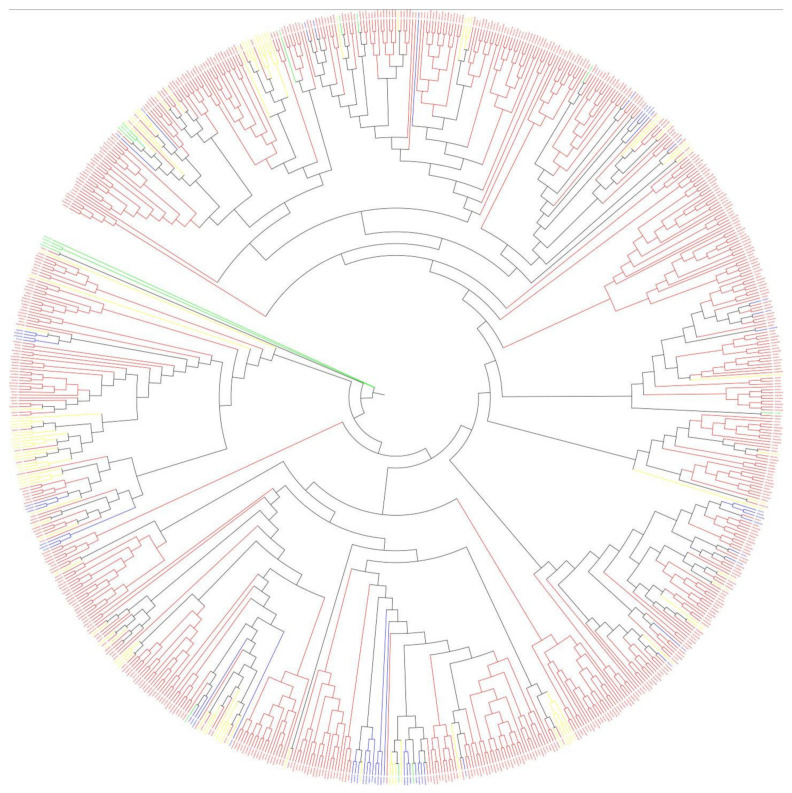
Phylogenetic tree of the 2OGD genes in *Brassica* species and *A. thaliana*. The DOXA class, DOXB class, DOXC class, and Unclassified are coloured blue, yellow, red, and green, respectively.

Phylogenetic analysis of each clade indicated that the functional diversity and phylogenetic distribution of 2OGD genes across plant species were related (Figure 5). The detailed tree is shown in Supplementary Figure S3. Although the three groups had very different amino acid sequences, the MP (maximum parsimony) method could still be used to construct a phylogenetic tree. Three classes of genes were scattered on different evolutionary branches, and some of them were close together, indicating that they had high homology and similar structures and functions (Figure 5). For example, the branches BnaC09g14250D- Bra027231.1- Bo5g126900.1- Bra027230.1- AT4G03070 may have high homology and similar structures and functions. According to these evolutionary branches, similar functions of the 2OGD genes in the three *Brassica* species could be inferred, but further research is needed to clarify gene function. At present, *A. thaliana* has done a lot of experiments to verify the related 2OGD gene function. They are involved in biosynthesis of gibberellin, gibberellin catabolism, glucosinolate metabolism, coumarin biosynthesis, salicylic acid catabolism, flavonoid metabolism, ethylene biosynthesis, etc. (See Supplementary Table S5). Specific *A. thaliana* 2OGD genes corresponded to the list of the three *Brassica* plant 2OGD homologous genes, and the corresponding predicted functions are shown in Supplementary Table S5. The functional prediction of these homologous genes is beneficial for us to provide a basis for further research on the 2OGD family of the three *Brassica* plant.

### 3.4. Gene Structure and Domain Analysis of the 2OGD Gene Family

**Figure 6:**
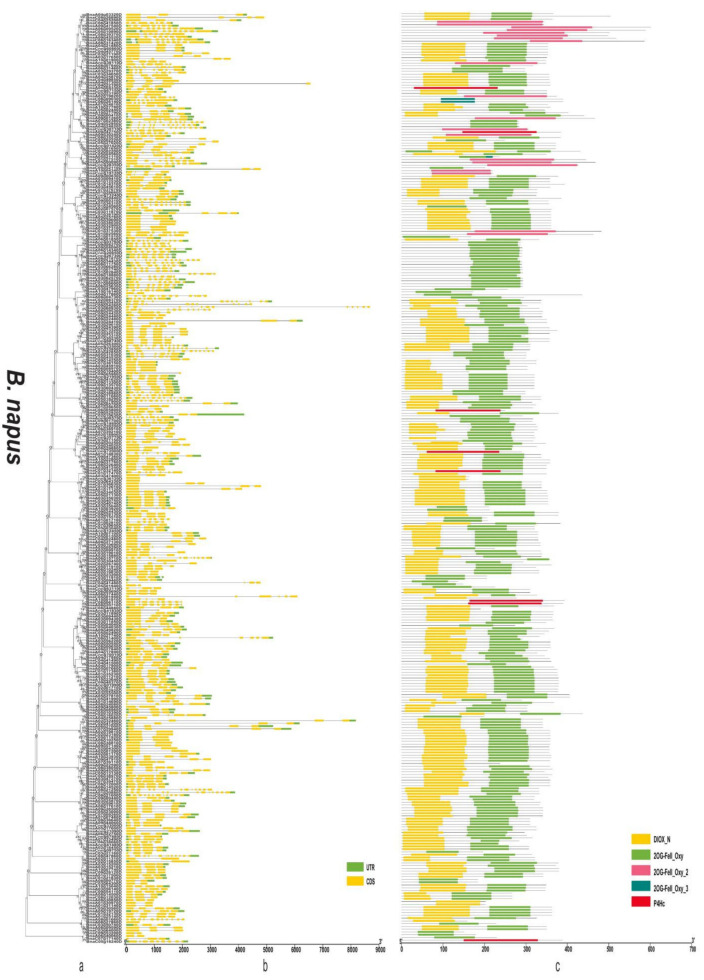
Phylogenetic relationships, gene structure, and domains in the 2OGD genes from *B. napus*. (**a**) The phylogenetic tree was constructed based on the full-length protein sequences of 2OGDsusing MEGA-X software. (**b**) Exon-intron structure of the 2OGD genes. Green boxes indicate untranslated 5′- and 3′- regions; yellow boxes indicate exons; and black lines indicate introns. (**c**) The domain composition of the 2OGD proteins. The domains DIOX_N and 2OG-FeII_Oxy. Some genes that only have 2OG-FeII_Oxy_2, 2OG-FeII_Oxy_3, or P4Hc are displayed in different coloured boxes. The length of proteins can be estimated using the scale at the bottom.

**Figure 7:**
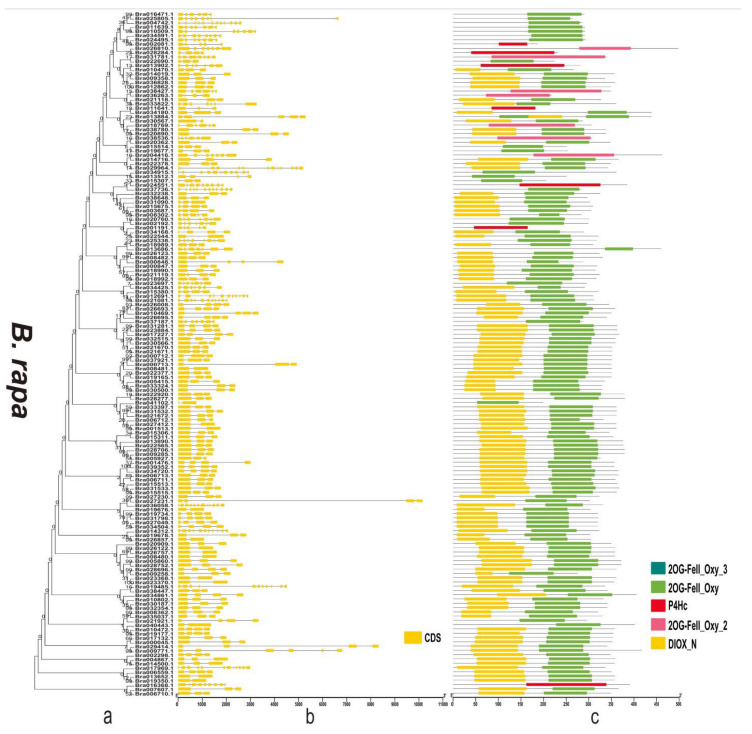
Phylogenetic relationships, gene structure, and domains in the 2OGD genes from *B. rapa*. (**a**) The phylogenetic tree was constructed based on the full-length protein sequences of 2OGDsusing MEGA-X software. (**b**) Exon-intron structure of the 2OGD genes. Green boxes indicate untranslated 5′- and 3′- regions; yellow boxes indicate exons; and black lines indicate introns. (**c**) The domain composition of the 2OGD proteins. The domains DIOX_N and 2OG-FeII_Oxy. Some genes that only have 2OG-FeII_Oxy_2, 2OG-FeII_Oxy_3, or P4Hc are displayed in different coloured boxes. The length of proteins can be estimated using the scale at the bottom.

**Figure 8:**
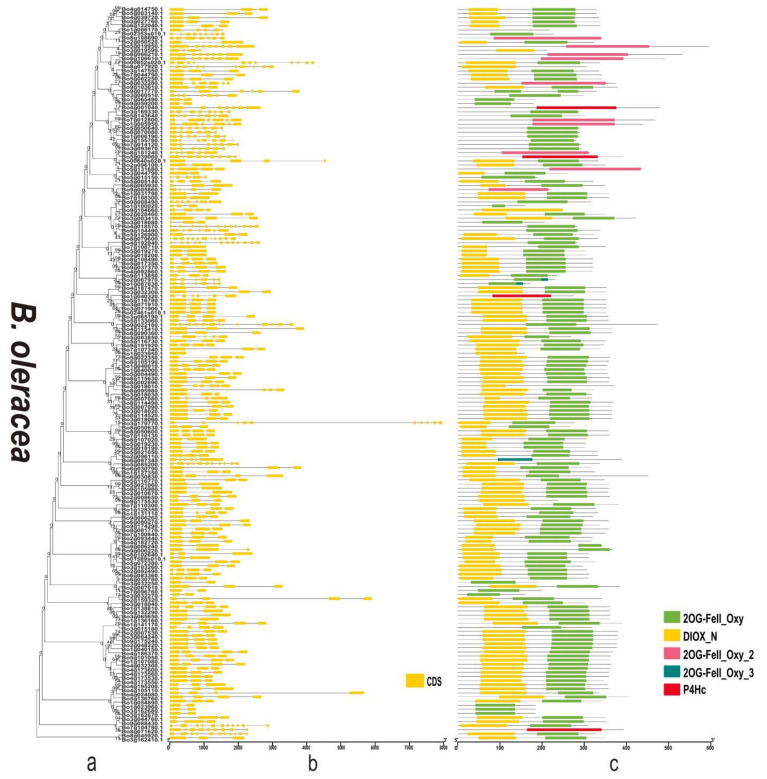
Phylogenetic relationships, gene structure, and domains in the 2OGD genes from *B. oleracea*. (**a**) The phylogenetic tree was constructed based on the full-length protein sequences of 2OGDs using MEGA-X software. (**b**) Exon-intron structure of the 2OGD genes. Green boxes indicate untranslated 5′- and 3′- regions; yellow boxes indicate exons; and black lines indicate introns. (**c**) The domain composition of the 2OGD proteins. The domains DIOX_N and 2OG-FeII_Oxy. Some genes that only have 2OG-FeII_Oxy_2, 2OG-FeII_Oxy_3, or P4Hc are displayed in different coloured boxes. The length of proteins can be estimated using the scale at the bottom.

By analysing the protein sequences of the *Arabidopsis* and three *Brassica* species 2OGDs and constructing rootless phylogenetic trees, their evolutionary relationships were analysed (Supplementary Figure S4a and Figure 6a, Figure 7a and Figure 8a). We generated exon-intron maps of the 2OGD genes based on their genome and coding sequences to better understand their structures. Gene structure analysis showed that there were approximately 1~12 exons in the 2OGD genes; for example, BnaA08g27860D, BnaC03g64330D, Bo7g108710.1, and Bo1g023950.1 have only one exon and Bo4g018570.1, Bra036058.1, and BnaC04g52950D have 12 exons (Figure 6b, Figure 7b and Figure 8b). In *Arabidopsis*, there are genes that have the same gene structure but different functions (Glucosinolate metabolism: At4g03070-At4g03060-At4g03050, Supplementary Figure S4b and Supplementary Table S5). Additionally, there are also some genes have similar functions with the same gene structure (Flavonoid metabolism: At5g63590-At5g63600, Supplementary Figure S4b and Table S5). There are many genes have the same exons and introns. For example, AT4G25300.1-AT4G25310.1 and AT5G20400.1-AT5G20550.1 are duplicated genes that have the same exons and introns. Additionally, the tandemly duplicated genes AT5G20400.1-AT5G20550.1 have more highly conserved exon-intron structures than paired duplicated AT1G49390.1-AT5G20400.1. Similarly, there are many identical gene structures in the three *Brassica* species genes. For example, BnaCnng60860D-BnaC04g45770D, BnaA01g28660D-BnaC-nng73240D, BnaA03g35030D-BnaC03g40750D-BnaC03g40740D-BnaA03g35040D, Bra021670.1-Bra021671.1, Bra033324.1-Bra030500.1, Bo5g019270.1-Bo5g018200.1, and Bo3g071910.1-Bo3g071900.1-Bo02461s010.1 are duplicated genes, and they have the same exon and intron structures (Supplementary Figure S4b and Figure 6b, Figure 7b and Figure 8b).

To better reveal their domains, we generated a domain map of the 2OGD genes according to their putative domain data (Supplementary Figure S4c and Figure 6c, Figure 7c and Figure 8c). The Pfam tool was used to further analyse domains present in the 2OGD proteins. The 2OG-Fe(II) oxygenase superfamily contains members of the 2-oxoglutarate (2OG) and Fe(II)-dependent oxygenase superfamily. The 2OGD family includes the C-terminus of the prolyl 4-hydroxylase α subunit. The family also includes lysyl hydrolases, isopenicillin synthases, and AlkB. All 2OGDs shared the DIOX_N and 2OG-FeII_Oxy domains. Some genes only have 2OG-FeII_Oxy_2, 2OG-FeII_Oxy_3, or P4Hc. In *A. thaliana* and the three *Brassica* species, most of the predicted 2OGDs contain DIOX_N and2OG-FeII_Oxy domains, and only a few 2OGDs (such as AT3G46500.1, BnaC05g18240D, Bra016368.1, and Bo8g071620.1) contain a single DIOX_N, 2OG-FeII_Oxy, or P4Hc domain (Supplementary Figure S4c and Figure 6c, Figure 7c and Figure 8c). The comparative analysis of the 2OGD genes highlighted that the gene structure and domain compositions of the 2OGD genes have been relatively conserved during evolution of the *Brassica* species.

### 3.5. Differentially Expressed A. thaliana 2OGD Genes at Different Developmental Stages

We searched the expression levels of 117 2OGD genes at different developmental stages in *A. thaliana* eFP Browser (Supplementary Figure S5). The expression data showed that the 2OGDs showed variable expression levels at different developmental periods. We investigated the expression of *A. thaliana* 2OGDs at 47 developmental stages (Supplementary Figure S6). The 2OGD genes were classified into three groups: the high expression group of 2OGD genes from At1g62380 to At2g43080 (group I), the medium expression group of 2OGD genes from At1g12010 to At4g03070 (group II) and the low expression group of 2OGD genes from At3g13610 to At5g59530 (group III) (Supplementary Figure S6). In the high expression group, the expression levels of genes in different tissues at different developmental stages were generally high, and only a few periods or tissues had relatively low expression (for example At2g38240 and At5g05600). The medium and low expression groups had a high expression level in specific tissues at certain periods, such as the At1g12010 and At5g54000 genes in Seeds Stage 8 w/o Siliques, Seeds Stage 9 w/o Siliques, and Seeds Stage 10 w/o Siliques, which were very high (Supplementary Figure S6). In summary, each 2OGD gene showed a unique expression pattern. To clarify the function of these genes, further function verification is needed.

### 3.6. Transcriptome Analysis of Tissue-Specific Expression

The RNA-seq data (GSE30795) from *A. thaliana* tissues showed that the 2OGDs have similar expression patterns in two accessions (Col-0 and Can-0) with variable expression patterns in three investigated tissues (seedling, root, and floral bud) (Supplementary Figure S7). The maximum expression of these 2OGD genes was observed in root tissues. Some 2OGD genes in *A. thaliana*, such as AT1G05010, AT1G62380, AT3G19010 and AT4G02940, were expressed at higher levels in the three investigated tissues (Supplementary Figure S7). Some genes were not expressed in various tissues, such as AT4G35820, AT1G48980, AT2G06960 and AT4G03060 (Supplementary Figure S7).

In the 25 investigated anatomical parts in *A. thaliana* (Supplementary Figure S8), different 2OGD genes were specifically expressed in different tissues (data obtained from GENEVESTIGATOR). We observed the maximum expression in seeds (embryo and endosperm) and roots (root endodermis and quiescent centre protoplast, root cell, root epidermis atrichoblast, root endodermis and quiescent centre cell, root stele cell, lateral root cap and columella cell, maturation zone, elongation zone, and radicle tip). Some 2OGD genes in *A. thaliana*, such as AT5G12270, GA2OX1, FLS2, ATJRG21, GA20OX1, GA2OX2, and SRG1, were preferentially expressed in the inflorescence, flower, pistil and receptacle (Supplementary Figure S8). The AT3G49630, AT4G25310, and AT5G51310 genes in *A. thaliana* were maximally expressed in the lateral root cap and columella cell. AT1G15540, AT3G46480, AT1G48980, AT2G06960, KUOX1, GA2OX7, and GA20OX4 were basically not expressed in any of the 25 anatomical parts of *A. thaliana* (Supplementary Figure S8). All *A. thaliana* 2OGD gene alias comparisons are listed in Supplementary Table S6.

In the 105 investigated anatomical parts in *A. thaliana* (Supplementary Figure S9), we observed that different 2OGD genes was specifically expressed in different tissues (all sample data obtained from the Affymetrix *Arabidopsis* ATH1 Genome Array (de-fault) platform in GENEVESTIGATOR). Some 2OGD genes in *A. thaliana*, such as GA3OX3, P4H6, GA3OX4, AT1G28030, and AT1G04380, were preferentially expressed in the micropylar endosperm, peripheral endosperm, and chalazal endosperm. Some genes had higher expression levels in anatomical parts of the root, such as AT5G05600, A2G38240, and AT1G77330 (Supplementary Figure S9).

**Figure 9:**
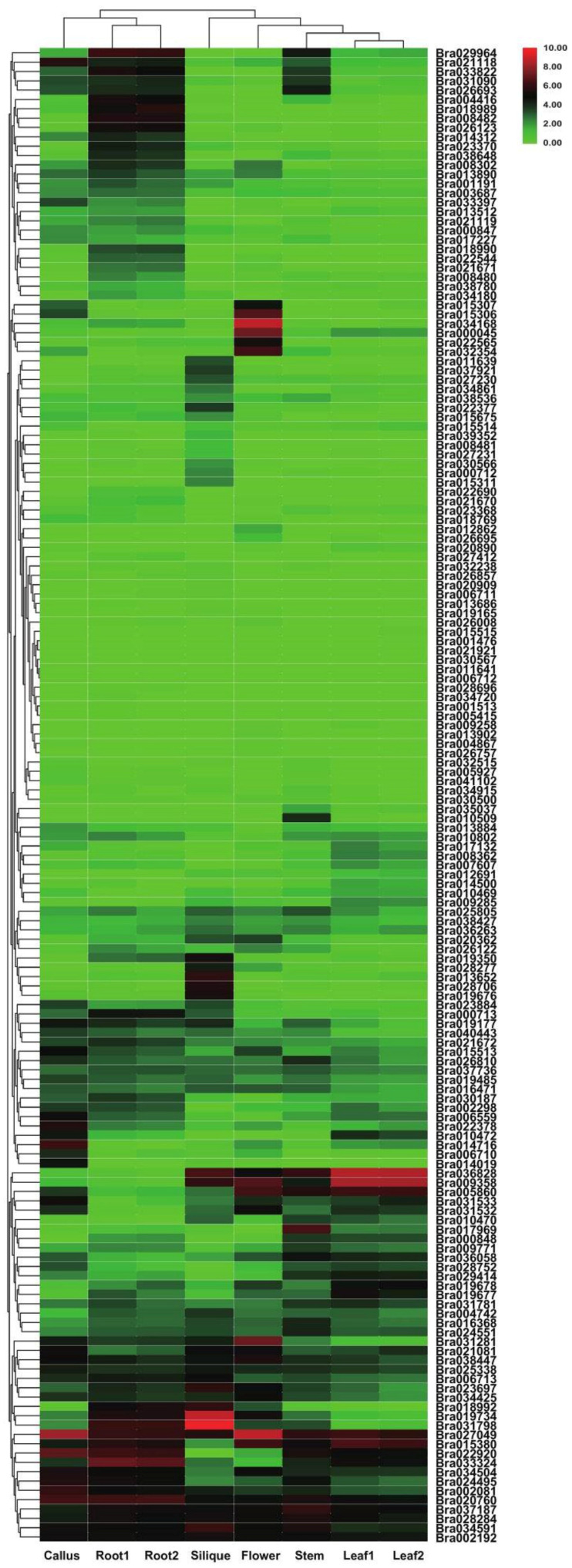
Tissue-specific expression of putative 2OGDs in *B. rapa*. Representative expression of putative 2OGDs of *B. rapa* in the callus, flower, root, leaf, and silique.

The RNA-seq data (GSE43245) from *B. rapa* tissues showed that 2OGDs had variable expression in all six investigated tissues (callus, flower, silique, leaf, root, and stem) (Figure 9). The maximum expression of these 2OGD genes was observed in the callus, root, silique and flower tissues. The expression level of some genes is high in a certain tissue, and it is suspected that it may play a key role in the 2OGDs family; for example, Bra019734, Bra031798, Bra027049, Bra034168 Bra036828, and Bra009358, etc. However, some genes are not expressed in various tissues, and it is speculated that these genes do not play an important role in the 2OGD family or even have no effect. For example, Bra015515, Bra001476, Bra021921, Bra030567, and Bra011641, etc. (Figure 9).

**Figure 10:**
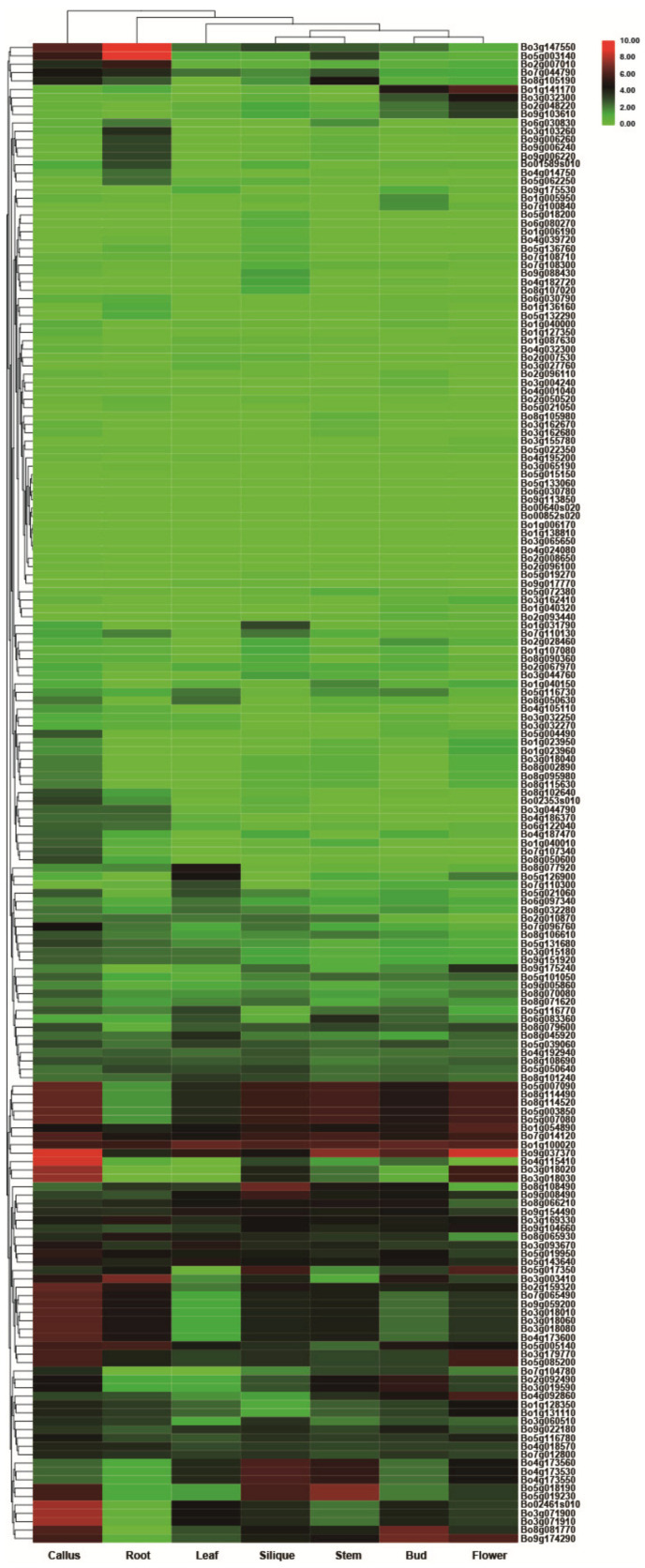
Tissue-specific expression of putative 2OGDs in *B. oleracea*. Representative expression of putative 2OGDs of *B. oleracea* in the callus, root, leaf, silique, stem, bud, and flower.

The RNA-seq data (GSE42891) from *B. oleracea* tissues showed that 2OGDs had variable expression in seven investigated tissues (callus, root, leaf, silique, stem, bud, and flower) (Figure 10). The maximum expression of these 2OGD genes was observed in the callus tissues. The expression level of some genes is high in a certain tissue, and it is suspected that it may play a key role in the 2OGDs family; for example, Bo3g147550, Bo5g003140, Bo9g037370, Bo4g115410 and Bo3g071900, etc. However, some genes are not expressed in various tissues, and it is speculated that these genes do not play an important role in the 2OGD family or even have no effect. For example, Bo3g065190, Bo5g015150, Bo5g133060, Bo6g030780, and Bo9g113850, etc. (Figure 10).

### 3.7. qRT-PCR Analysis in Different Tissues

Gibberellin (GA) is a plant hormone that regulates various developmental processes in plants like seed germination, stem elongation, flowering, and fruit ripening [17,39]. Therefore, we selected six Bo2OGD genes related to gibberellin synthesis and catabolism in Chinese kale for qRT-PCR analysis to see if these genes are involved in the regulation of these developmental processes. The expression patterns of the six Bo2OGDs gene in different tissues of Chinese kale were preliminarily analysed by qRT-PCR using fully grown plants (Supplementary Figure S1).

**Figure 11:**
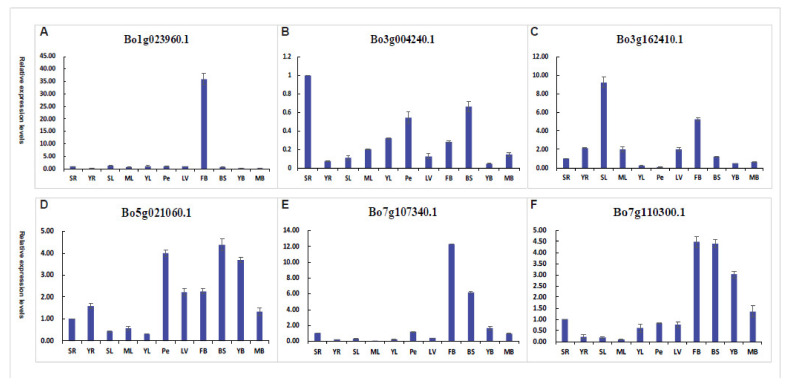
Tissue-specific qRT-PCR analysis of putative six Bo2OGD genes. (**A**–**F**) respectively represent the tissue-specific qRT-PCR analysis of a single Bo2OGD gene. Sample identifiers: senescent leaves (SL), mature leaves (ML), young leaves (YL), leaf veins (LV), petioles (Pe), young bolting stem flesh (YB), middle-aged bolting stem flesh (MB), bolting stem skin (BS), flower buds (FB), senescence roots (SR), and young roots (YR).

The expression levels of the Bo1g023960.1 in flower buds were higher more than thirty times those in other 10 tissues (Figure 11A), such as leaves, leaf veins, petioles, young bolting stem flesh, middle-aged bolting stem flesh, bolting stem skin, and roots. This suggests that the gene may be involved in the process of flowering. The expression level of Bo3g004240.1 is low in eleven tissues, and the value of the relative expression of the highest tissues is also 1 (Figure 11B). It may be that the gene does not play a significant role during this period. The expression levels of the Bo3g162410.1 in senescent leaves and flower buds were higher more than five times those in other nine tissues (Figure 11C). This suggests that the gene may be involved in the process of flowering and leaf senescence. In the Bo5g021060.1 gene, the relative expression of petiole, bolting stem skin, and young bolting stem flesh, and the relative expression of other tissues was lower (Figure 11D). The expression levels of the Bo7g107340.1 in flower buds and bolting stem skin were higher more than six times those in other nine tissues (Figure 11E). This suggests that the gene may be involved in the process of flowering and stem elongation. In the Bo7g110300.1 gene, the relative expression of flower buds, bolting stem skin, and young bolting stem flesh, and the relative expression of other tissues was lower (Figure 11F). This suggests that the gene may be involved in the process of flowering and stem elongation. In general, these six genes have different tissue specificities and play different roles in different parts.

## 4. Discussion

### 4.1. Evolution and Expansion of 2OGD Genes

*Brassica* is an important genus of Cruciferae and contains many common economic crops. Approximately 20 million years ago (MYA), *Brassica* and *A. thaliana* diverged from a common ancestor; then, approximately 16 MYA, *Brassica* ancestors underwent a whole genome triplication (WGT) event [40]. Phylogenetic analysis divided the species of *Brassica* into *B. rapa*, *B. oleracea*, and *B. nigra* lineages, which diverged approximately 8 MYA [41]. In *Brassica* plants, the interspecific cytogenetic relationship between important crops is well described with a “U” triangle, with two diploid species each (*B. rapa* (AA, n = 10), *B. oleracea* (CC, n = 9), and *B. nigra* (BB, n = 8)) forming a tetraploidy species (*B. napus* (AACC, n = 19), *B. juncea* (AABB, n = 18), or *B. carinata* (BBCC, n = 17)) [42,43]. With the complete genome sequencing of *B. rapa*, *B. oleracea*, and *B. napus*, great progress has been made in the study of the origin and evolution of *Brassica* plants. The higher number of putative 2OGDs (337) identified in *B. napus*, compared to its diploid progenitors *B. rapa* (160 2OGDs) and *B. oleracea* (179 2OGDs), demonstrates the direct effect of polyploidization in increasing the number of 2OGDs in polyploid crops (Supplementary Table S1). The number of 2OGDs identified in haploid crops was basically the same (130 in *A. thaliana* [27], 114 in *O. sativa* [28], 142 in *P. abies* [29], and 132 in *S. miltiorrhiza* [30]). Therefore, we speculate that the biosynthesis of secondary metabolites in polyploid crops (tobacco, wheat, cotton, peanuts, apples, etc.) is diverse due to the increase in the number of 2OGD genes.

Plants have experienced more genome duplication events than any other eukaryotes on the earth. After the duplication, genes can follow one of the three functional outcomes, gene loss, neo-functionalization and sub-functionalization [44,45]. In duplication events, either it is whole genome, segmental or tandem, some genes have more probability to be retained as duplicates. This may be attributed to the certain function of the duplicated genes in particular organism [45,46,47]. In the chromosomal distribution analysis, three *Brassica* plants had a varied distribution of 2OGD genes. In the comparisons of the *B. rapa* AA genome to the *B. napus* AA genome and the *B. oleracea* CC genome to the *B. napus* CC genome, the genomes shared the same pattern of chromosomes, reflecting the occurrence of recent gene duplication events and close phylogenetic relationships. This is consistent with the findings for *O. sativa* [28], *A. thaliana* [27], *P. abies* [29], and *S. miltiorrhiza* [30] 2OGDs. Tandemly duplicated 2OGD genes in *B. rapa*, *B. napus*, and *B. oleracea* suggest that chromosomal rearrangements participated in the versatility of plant secondary metabolism. The exon-intron structure of the 2OGD gene map (Figure 6b, Figure 7b, and Figure 8b) shows an increased number of homologous 2OGD gene pairs in *B. napus* compared to *B. rapa* and *B. oleracea*, suggesting that polyploidization increased the number of homologous genes during the hybridization of genomes. Interestingly, we also found in the study by Rehman et al. [48] that the three *Brassica* species UGTs (UDP-glycosyltransferases) families also have gene duplication and clustering. Their genomes share the same chromosome pattern, and the number of gene families is also related to poly-ploidization (They identified 140, 154, and 251 putative UGTs in *B. rapa*, *B. oleracea*, and *B. napus*, respectively). Similarly, a previous study identified 48, 35, and 92 putative GRAS (gibberellic acid insensitive, repressor of GAI, and scarecrow) in *B. rapa*, *B. oleracea*, and *B. napus*, respectively [49]. Lohani et al. [50] identified 36, 35, and 64 putative Hsf (Heat stress transcription factors) in *B. rapa*, *B. oleracea*, and *B. napus*, respectively. Thus, we speculate that the evolutionary characteristics of the other gene families of these three *Brassica* species will have similar patterns.

The 2OGD superfamily is divided into DOXA, DOXB and DOXC, which function differently. The DNA repair protein of *Escherichia coli* AlkB is induced by responding to alkylating agents [51]. AlkB belongs to the DOXA class of 2OGDs. The key enzymes for mammalian collagen synthesis and hypoxic signalling are prolyl 4–hydroxylases (P4Hs) [52]. When plants and algae synthesize cell wall proteins, P4H is also involved in its synthesis [12,53], and belongs to the DOXB class of 2OGDs. The 2OGD genes that function in antibiotic biosynthesis, functional collagen biosynthesis and stress responses (such as flavonoid metabolism, gibberellin biosynthesis and catabolism, auxin metabolism, ethylene biosynthesis, and glucose metabolism) belong to the DOXC class of 2OGDs. DOXA and DOXB are universally widespread in metabolism and they are involved in nucleotide and protein modification, respectively [1,8]. Conversely, the diversity of DOXC class 2OGDs is due to lineage-specific metabolism in plants. By comparing four plant model genomes, we revealed the vast functionality, diversity and evolution of plant 2OGDs. Notably, the number of DOXA and DOXB 2OGDs was similar for the four plant taxa, whereas the number of DOXC 2OGDs was about seven times more than them (Figure 4).

The 2OGD genes of *A. thaliana* are involved in various biosynthesis and metabolism enzymes, and these genes have evident replication and proliferation in *Brassica* plants. Gene multiplication of these metabolic enzymes followed by functional differentiation of their paralogs was probably the driving force for producing specialized metabolites adapted to the particular environments where *Brassica* plants. The emergence of a large number of diverse metabolites also confirms that the 2OGD gene family is constantly evolving and is involved in the synthesis and metabolic assembly of these metabolites. It needs further study to analyse the functions of 2OGD gene in *B. rapa*, *B. oleracea*, and *B. napus*, to find whether the functional differentiation exists and to explore their precise bio-logical roles.

### 4.2. Gene Expression and Transcriptome Analysis Could Better Predict Potential Gene Functions

From the analysis of the expression of 2OGDs in different developmental stages of *A. thaliana*, the expression levels of the 2OGD genes were different at different developmental stages. The 2OGD genes in groupⅠ were expressed at higher levels in all different developmental stages, and the 2OGD genes in groupⅡ, Ⅲ were expressed higher only at a specific developmental stage (Supplementary Figure S6). We found that At1g12010 and At4g03050 are highly expressed in the seed stage and low in other growth stages, and this suggested that At1g12010 may be involved in ethylene synthesis [54] and At4g03050 involved in glucosinolate metabolism [23]. Therefore, we speculated that genes clustered together with the same expression patterns may have similar functions, namely At3g11180, At1g04380, At1g28030, and At4g10490. Some 2OGD genes in groupⅡ, Ⅲ, were expressed relatively low in some stages, and some genes were not expressed at all stages of development (Supplementary Figure S6), which suggested that At4g35820, At5g07480, and At2g44800 may be not important genes involved in the metabolism process. These results indicate that there are key different 2OGD genes at different developmental stages that regulate plant secondary metabolism. This provides a basis for the future study of plant secondary metabolism.

Interestingly, their expression patterns are the same in the results from the transcriptomes of the seeds, roots, and flower buds of the two ecotypes of *A. thaliana* (Supplementary Figure S7). From the analysis of transcriptome data of different organs and tissues, it was found that among the 130 2OGD genes of *A. thaliana*, there were 35 genes with higher relative expression in each tissue, while other genes were highly expressed in a specific tissue. For example, AT1G05010 is highly expressed in seeds, roots, and flower buds, especially in seeds. We speculate that this gene plays an important role in the entire growth and development of *A. thaliana* and is related to the regulation of fruit ripening according to a previous study, which found that AT1G05010 is involved in ethylene biosynthesis [54]. Some genes had low or no expression in any tissue (Supplementary Figure S7), for example, AT4G22870, AT3G47190, AT2G44800, AT1G48980, AT2G06960, AT1G17010, etc. It is speculated that these genes are not important genes involved in the metabolism of the 2OGD family, for example, AT4G22870, AT3G47190, AT2G44800, AT1G48980, AT2G06960, and AT1G17010, etc. It is speculated that these genes are not important genes involved in the metabolism process. In addition, we found that only one gene (AT2G25450) showed a different expression pattern. It is highly expressed in seeds, roots, and flower buds in Col-0 *A. thaliana*, while it is not expressed in Can-0 *A. thaliana*, which is according with the results that AT2G25450 is involved in 2-hydroxybut-3-enyl glucosinolate biosynthesis [24] in Col-0 *A. thaliana*, but not in Can-0 *A. thaliana*.

*B. rapa* and *B. oleracea* had similar expression patterns. A small number of genes were highly expressed in various tissues, other genes were highly expressed in a specific tissue, other genes were expressed at a very low level, and some genes in the individual tissues were not expressed (Figure 9 and Figure 10). According to the heat map of tissue-specific 2OGD genes in *B. rapa* (Figure 9), Bra015307, Bra015306, Bra034168, Bra000045, Bra022565, and Bra032354 had similar expression patterns. They were highly expressed in flower and low in other tissues. We speculated that these genes may be involved in the gibberellin metabolism for regulating plant flowering process. Bra019734, and Bra031798 are highly expressed in silique and low in other tissues, which suggested that these two genes may be involved in ethylene biosynthesis during fruit ripening. According to the heat map of tissue-specific 2OGD genes in *B. oleracea* (Figure 10), Bo1g054890, Bo7g014120, Bo1g100020, and Bo9g037370 were highly expressed in callus, root, leaf, silique, stem, bud, and flower, which suggested that these genes may be important genes in regulating secondary metabolism at different development stages.

By qRT-PCR analysis of different tissue parts, it was found that Bo1g023960.1 and Bo7g107340.1 have high relative expression in flower buds, while other tissues have low expression levels (Figure 11). Because these two genes are homologous genes of Arabidopsis At4g21200 (GA2ox8), according to the known altered expression of GA2ox7 and GA2ox8 affects flowering behaviour [55], we speculate that these two genes have the regulation process of gibberellin to regulate plants flowering function. Bo3g004240.1, Bo5g021060.1, and Bo7g110300.1 have relatively high expression in bolting stem skin, petioles, young bolting stem flesh or middle-aged bolting stem flesh, while other tissues have low expression levels (Figure 11). As these genes are homologous genes of *A. thaliana* At5g07200 (GA20ox3), At1g15550 (GA3ox1), At4g25420 (GA20ox1), and they play roles in stem elongation [56,57,58,59], we speculated that these genes may be involved in the process of gibberellin metabolism f in stem growth. Although these speculations need further experiments to verify, but these data also provide an important basis for researchers interested in the function of these genes.

Gene function has been largely identified based on the 2OGD genes in *A. thaliana* (Supplementary Table S5), such as participation in biosynthesis of gibberellin, gibberellin catabolism, flavonoid metabolism, ethylene biosynthesis, glucosinolate metabolism, coumarin biosynthesis, and salicylic acid catabolism [1]. However, the known gene functions of the 2OGD gene family in these three species of *Brassica* is relatively limited, and further research is needed on each gene.

## 5. Conclusions

In this study, a comprehensive analysis of the 2OGD gene family in three *Brassica* plants was performed. We identified 676 2OGD genes from three *Brassica* plants and analysed their chemical characteristics, structures, sequence homologues, phylogenetic relationships, tandem duplication and expansion, protein structure domains, transcriptome and expression analyses. Additionally, based on the function of known *A. thaliana* 2OGD gene, their homologous 2OGD gene function was predicted (Supplementary Table S5). This lays the foundation for the evaluation of the 2OGD gene family and the basis of future functional studies. In the future, functional in-depth studies of certain important 2OGD genes (such as genes participation in biosynthesis of gibberellin, gibberellin catabolism, flavonoid metabolism, ethylene biosynthesis, glucosinolate metabolism, coumarin biosynthesis, and salicylic acid catabolism, etc.) are needed to reveal the complexity of the 2OGD gene family in the biological processes of organisms. Our results provide a valuable resource for better understanding the biological roles of individual 2OGD genes in *Brassica* plants.

## Supplementary Materials

The following are available online at https://www.mdpi.com/article/10.3390/genes12091399/s1, Figure S1: Different tissues used in qRT-PCR analysis. Sample identifiers: senescent leaves (SL), mature leaves (ML), young leaves (YL), leaf veins (LV), petioles (Pe), young bolting stem flesh (YB), middle-aged bolting stem flesh (MB), bolting stem skin (BS), flower buds (FB), senescence roots (SR), and young roots (YR); Figure S2: Chromosomal distribution of putative 2OGDs in *A. thaliana*. Individual 2OGD positions are shown in Mbs along the chromosome scale; Figure S3: Phylogenetic tree of the 2OGD genes in *Brassica* species and *A. thaliana*. The DOXA class, DOXB class, DOXC class, and Unclassified are coloured blue, yellow, red, and green, respectively; Figure S4: Phylogenetic relationships, gene structure and domains in the 2OGD genes from *A. thaliana*. (a) The phylogenetic tree was constructed based on the full-length protein sequences of 2OGDsusing MEGA-X software. (b) Exon-intron structure of the 2OGD genes. Green boxes indicate untranslated 5′- and 3′- regions; yellow boxes indicate exons; and black lines indicate introns. (c) The domain composition of the 2OGD proteins. The domains DIOX_N and 2OG-FeII_Oxy. Some genes that only have 2OG-FeII_Oxy_2, 2OG-FeII_Oxy_3, or P4Hc are displayed in different coloured boxes. The length of proteins can be estimated using the scale at the bottom; Figure S5: *A. thaliana* different developmental stages map; Figure S6: Differential expression of 2OGDs in different *Arabidopsis* developmental periods. The green colour shows low-expression 2OGDs, and the red colour shows high-expression 2OGDs; Figure S7: Tissue-specific expression of putative 2OGDs in *A. thaliana*. Representative expression of *A. thaliana* 2OGDs in seedlings, roots, and floral buds; Figure S8: Representative expression of *A. thaliana* 2OGDs in 25 various tissues; Figure S9: Representative expression of *A. thaliana* 2OGDs in 105 different anatomical parts; Table S1: List of all 2OGD genes; Table S2: Primers used in the qRT-PCR; Table S3: The identification of homologous 2OGD members; Table S4: The cellular localization prediction of 2OGD superfamily members; Table S5: The function prediction of 2OGD superfamily members in three *Brassica* plants; Table S6: *A. thaliana* 2OGD gene alias comparison.
