# Supplementary figures and images for: Genome-Wide Identification and Expression Profiling of 2OGD Superfamily Genes from Three Brassica Plants
Source: Genes (Basel). 2021 Sep 10;12(9):1399. doi: 10.3390/genes12091399 (PMC8465909; doi:10.3390/genes12091399)

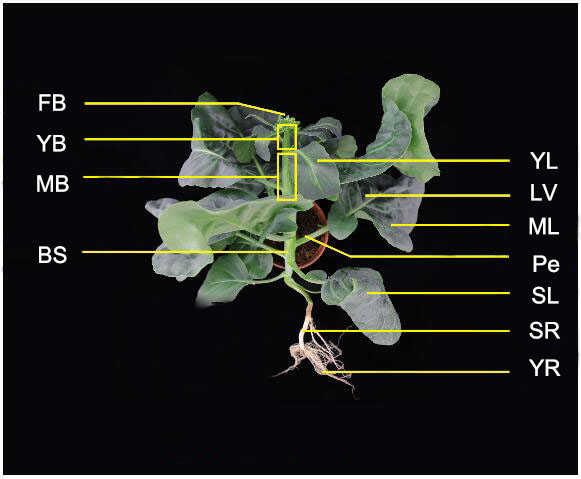

Supplement: Supplementary file 1 [file genes-12-01399-s001.zip › Supplementary_Figure_1.tif]

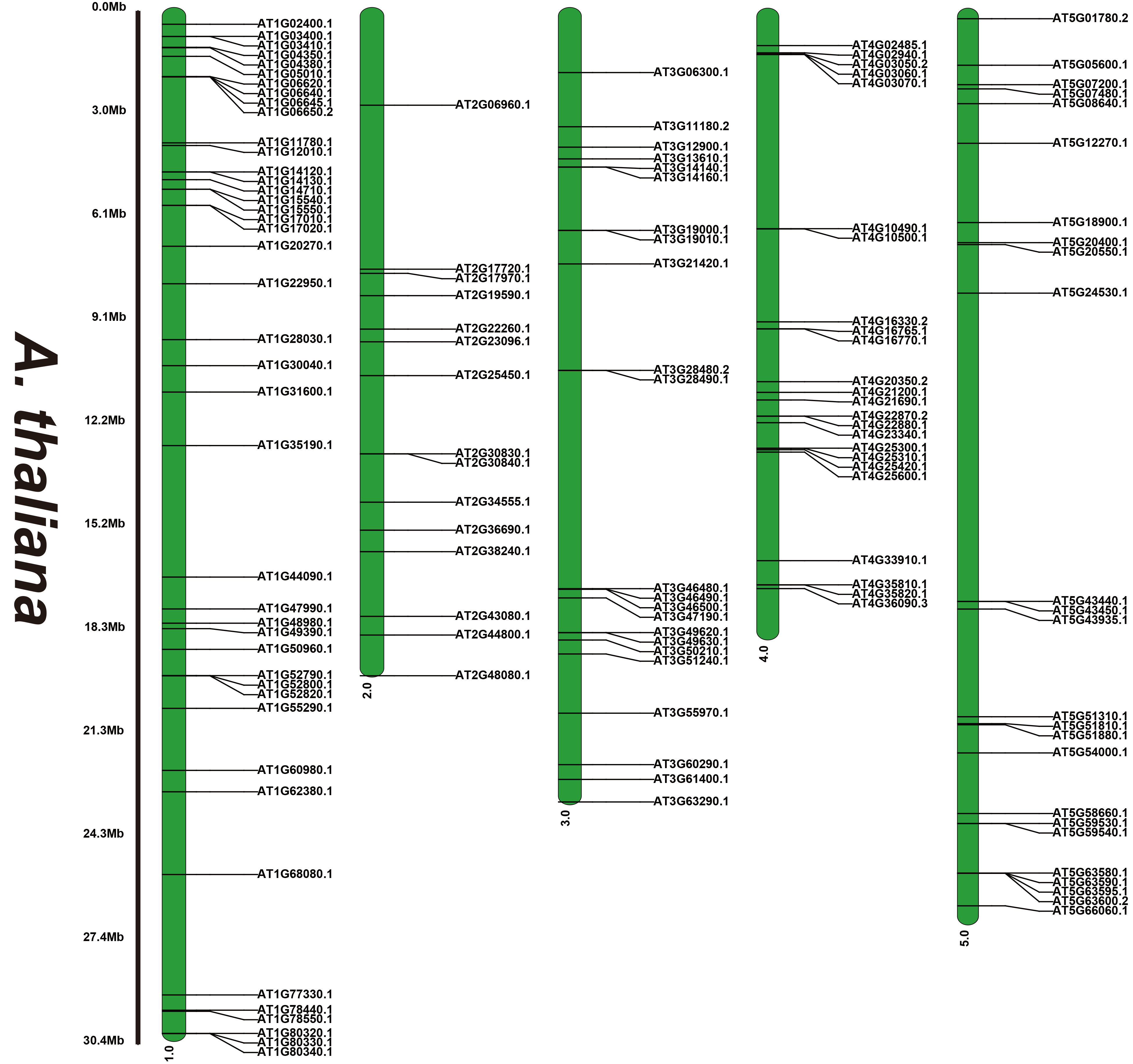

Supplement: Supplementary file 1 [file genes-12-01399-s001.zip › Supplementary_Figure_2.tif]

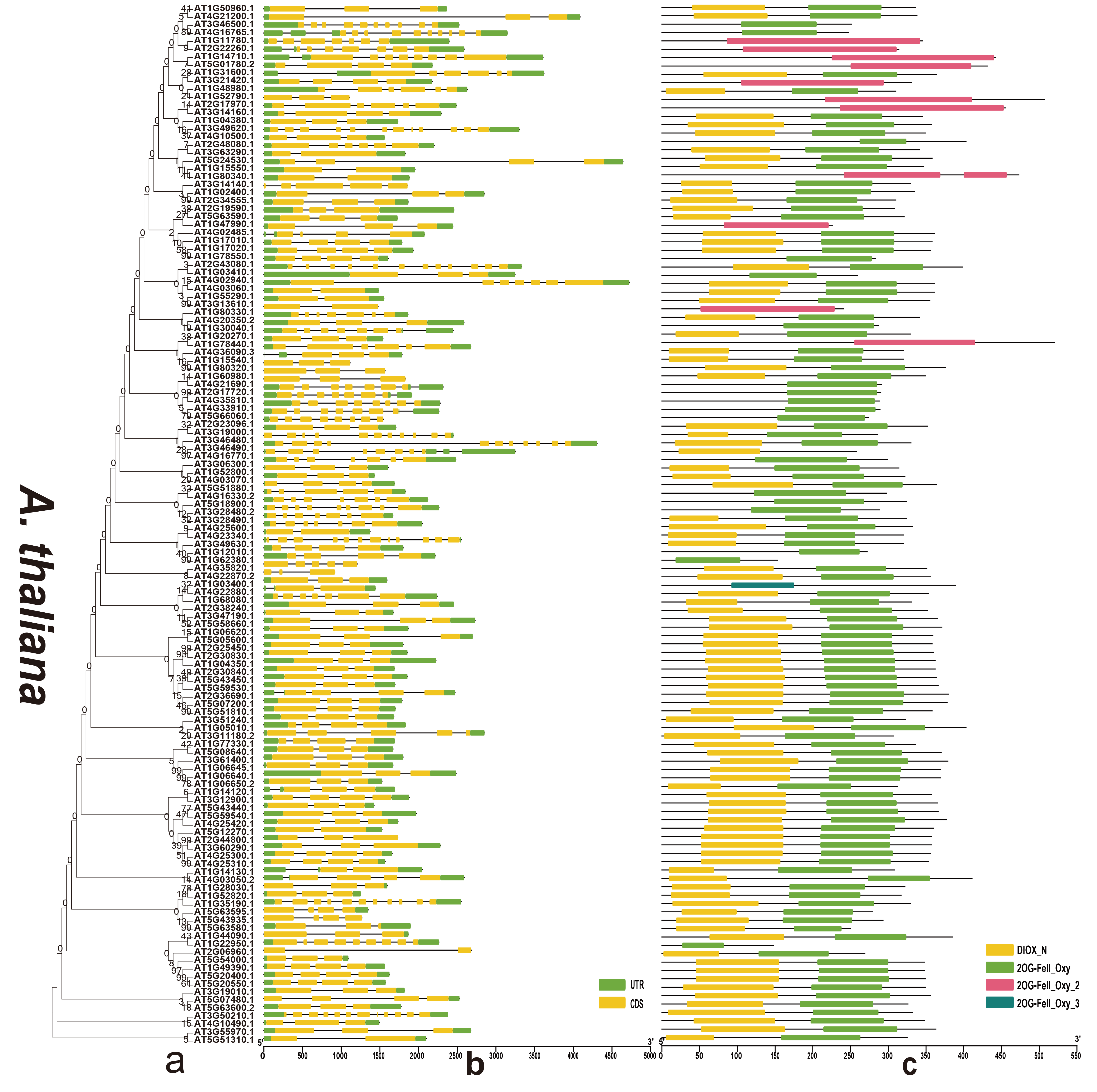

Supplement: Supplementary file 1 [file genes-12-01399-s001.zip › Supplementary_Figure_4.tif]

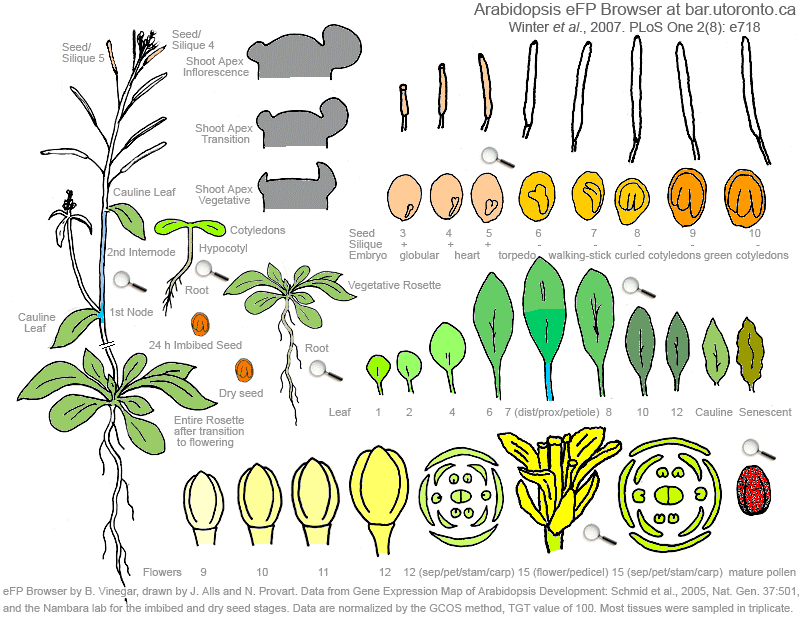

Supplement: Supplementary file 1 [file genes-12-01399-s001.zip › Supplementary_Figure_5.tif]

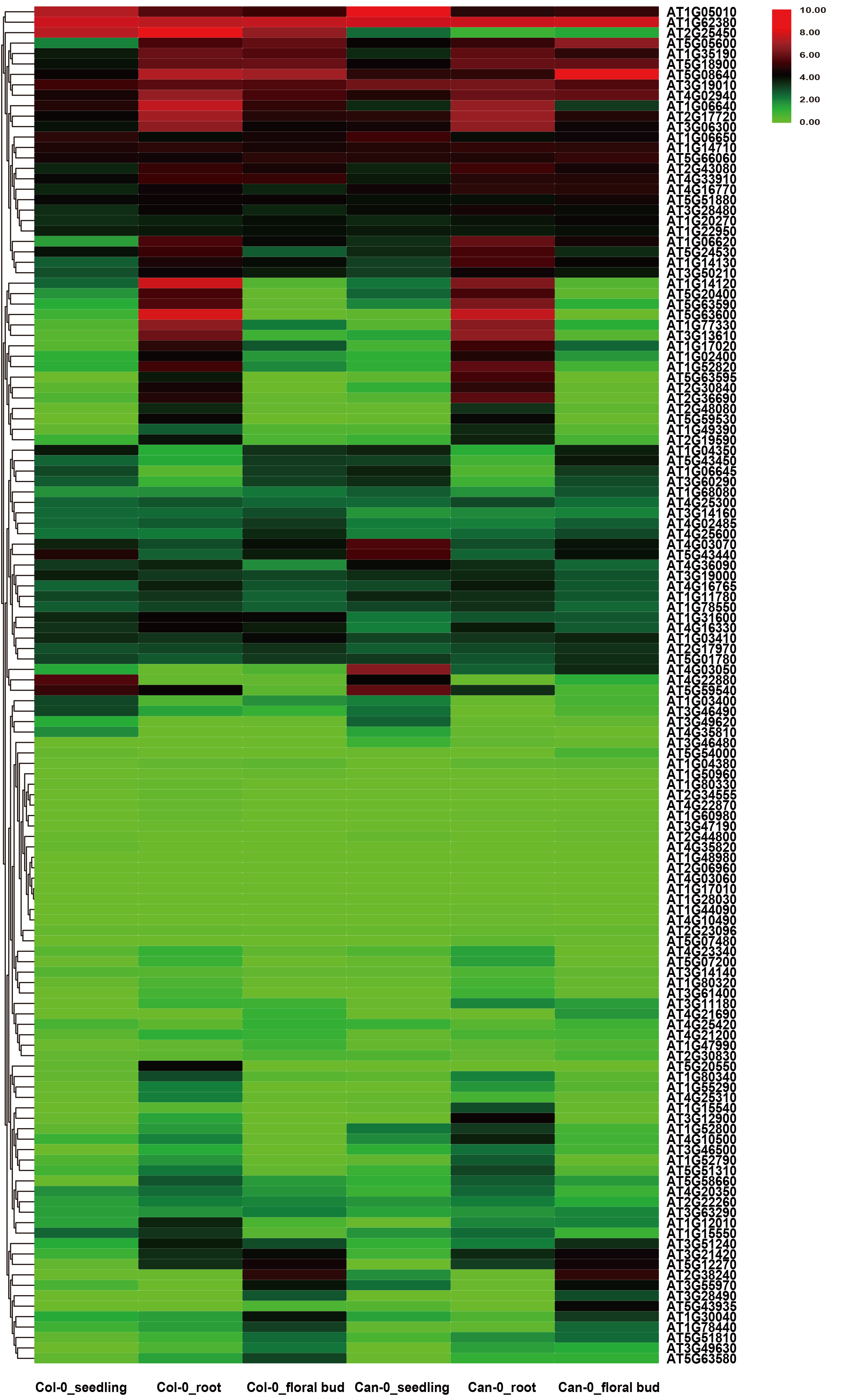

Supplement: Supplementary file 1 [file genes-12-01399-s001.zip › Supplementary_Figure_7.tif]

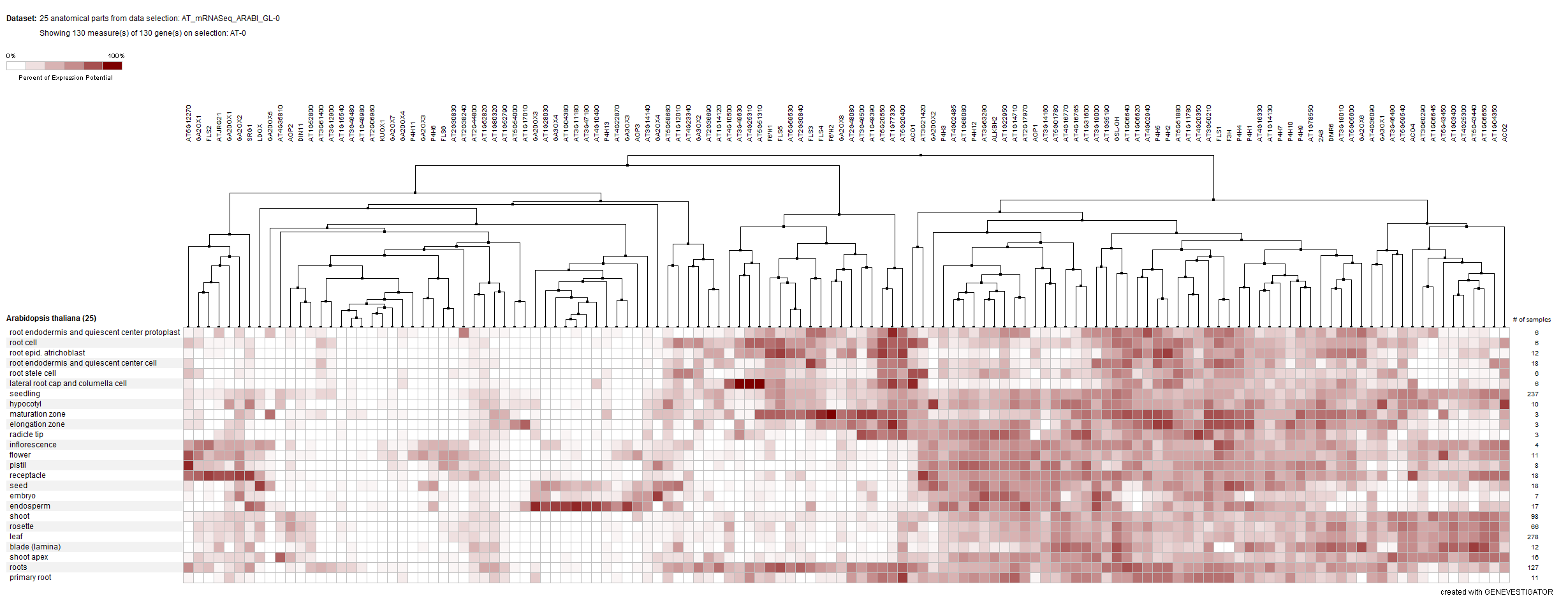

Supplement: Supplementary file 1 [file genes-12-01399-s001.zip › Supplementary_Figure_8.tif]

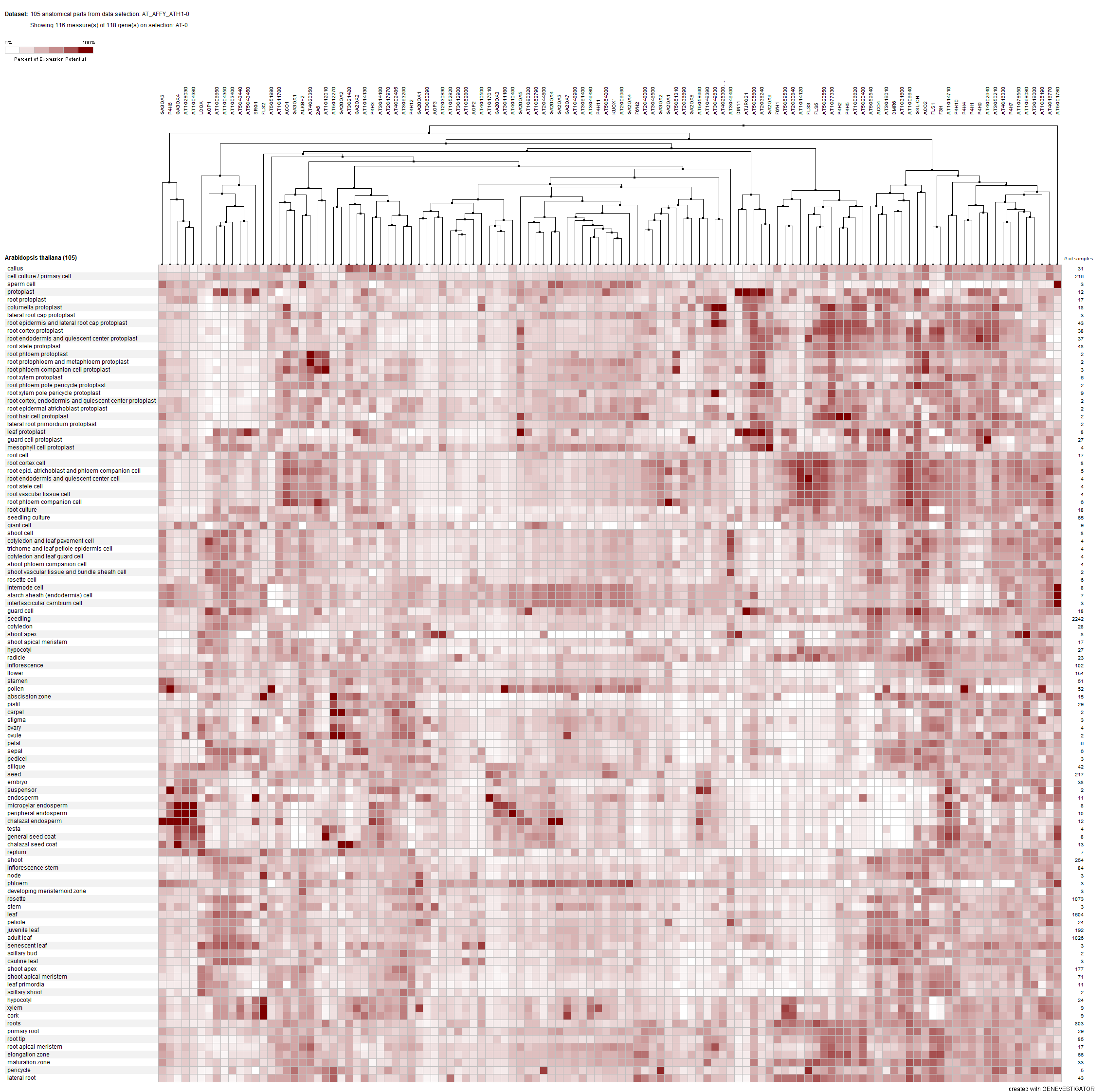

Supplement: Supplementary file 1 [file genes-12-01399-s001.zip › Supplementary_Figure_9.tif]
