# Supplementary material for: Genome-Wide Identification and Expression Profiling of 2OGD Superfamily Genes from Three Brassica Plants
Source: Genes (Basel). 2021 Sep 10;12(9):1399. doi: 10.3390/genes12091399 (PMC8465909; doi:10.3390/genes12091399)

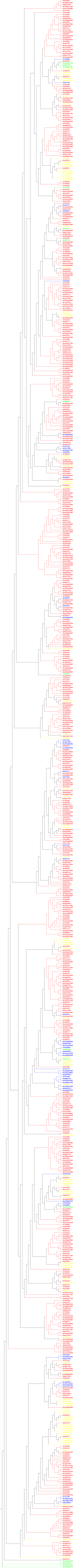

Supplement: Supplementary file 1 [file Supplementary_Figure_3.pdf › Supplementary_Figure_3.pdf]
